# Computational enzymology for degradation of chemical warfare agents: promising technologies for remediation processes

**DOI:** 10.3934/microbiol.2017.1.108

**Published:** 2017-03-14

**Authors:** Alexandre A. de Castro, Letícia C. Assis, Daniela R. Silva, Silviana Corrêa, Tamiris M. Assis, Giovanna C. Gajo, Flávia V. Soares, Teodorico C. Ramalho

**Affiliations:** 1 Department of Chemistry, Federal University of Lavras, 37200-000, Lavras, Brazil; 2 Center for Basic and Applied Research, Faculty of Informatics and Management, University of Hradec Kralove, Rokitanskeho 62, 50003, Czech Republic

**Keywords:** enzymatic biodegradation, warfare nerve agents, computational methods, oximes

## Abstract

Chemical weapons are a major worldwide problem, since they are inexpensive, easy to produce on a large scale and difficult to detect and control. Among the chemical warfare agents, we can highlight the organophosphorus compounds (OP), which contain the phosphorus element and that have a large number of applications. They affect the central nervous system and can lead to death, so there are a lot of works in order to design new effective antidotes for the intoxication caused by them. The standard treatment includes the use of an anticholinergic combined to a central nervous system depressor and an oxime. Oximes are compounds that reactivate Acetylcholinesterase (AChE), a regulatory enzyme responsible for the transmission of nerve impulses, which is one of the molecular targets most vulnerable to neurotoxic agents. Increasingly, enzymatic treatment becomes a promising alternative; therefore, other enzymes have been studied for the OP degradation function, such as phosphotriesterase (PTE) from bacteria, human serum paraoxonase 1 (*Hss*PON1) and diisopropyl fluorophosphatase (DFPase) that showed significant performances in OP detoxification. The understanding of mechanisms by which enzymes act is of extreme importance for the projection of antidotes for warfare agents, and computational chemistry comes to aid and reduce the time and costs of the process. Molecular Docking, Molecular Dynamics and QM/MM (quantum-mechanics/molecular-mechanics) are techniques used to investigate the molecular interactions between ligands and proteins.

## 1. Introduction

Chemical weapons are part of a group of mass destruction weapons that represent a major threat to society. They constitute a non-conventional class of low-cost, with difficult detection and control [1]. In this context, organophosphorus compounds (OP) are organic compounds quite used in the fight against pests in agriculture, because they are relatively cheap and present activity on different pests [2],[3]. However, they are very dangerous because of their neurotoxic effects which can cause serious damage to health public and environment [4],[5]. In fact, the use of toxic and warfare nerve agents has been reported since antiquity, and the most recent employ of this kind of weapon took place in Syria, on August 21, 2013, where there was an attack with the neurotoxic agent Sarin (OP derivative), resulting in more than 3,600 victims [6]. Due to the wide use of these agents, the current concern is to design effective antidotes for the poisoning caused by them [4].

The enzymatic treatment has become a promising alternative because conventional methods of OP detoxification are expensive and require severe conditions, while enzymes are known for their diversity and ability to perform chemical reactions at high speed rates [7],[8]. Research areas commonly include enzyme catalysis, related to protein stability, stereoselectivity and substrate preference [9]. Another treatment process is performed by employing certain antidotes, the so-called oximes. In this case, the treatment for OP poisoning consists in the removal of the phosphoryl group from the Acetylcholinesterase (AChE) active site through the reactivation promoted by these compounds [10].

The methods and techniques used in computational chemistry can be very useful to understand the mechanisms by which the enzymes act and how this occurs at the atomic level, since it reduces the costs and time of research [11]. Among the most used methods, we can cite Molecular Docking (which calculates the best fit of ligands in a protein active site), Molecular Dynamics and QM/MM (quantum-mechanics/molecular-mechanics), which can be used to investigate the molecular interactions between ligands and proteins [7]. In this line, this review aim to present some of the most important methods for the remediation of the intoxication caused by OP, and how these techniques can be studied and foreseen by computational calculations.

## 2. Materials and Method

### 2.1. Molecular docking

In recent years, the research involving computational chemistry methods and the enzymology field has been maturing. Since then, these computational tools can provide us important data on the understanding, at the atomic level, of the fundamental mechanisms that involve enzymatic catalysis [12],[13].

Currently, Molecular Docking is one of several computational methodologies widely used in the prediction of ligand conformation and its orientation within the protein interaction site [14]. In addition, it is an important tool for investigating the molecular interactions between protein and ligand in cases where the three-dimensional structure of the protein has already been elucidated [15]. There are currently several docking softwares, such as GOLD® [16], AUTODOCK [17],[18], FLEXX [19], MOLEGRO VIRTUAL DOCKER (MVD)® [15].

During a molecular docking simulation, many conformations are generated for the ligand in the protein active site. For this reason, it is necessary a model that allows the evaluation of all possible positions acquired by the ligand and its relative stability throughout the simulation, and thus choosing the most stable structure of the complex (protein-ligand). This selection model can be expressed by the combination of two components: a search algorithm and a scoring function [7],[15],[20].

The search algorithm analyzes the various degrees of freedom of the enzymatic side chains, and also the molecular structure of a small molecule by generating a conformation of the ligand and its orientation at the protein interaction site [7],[20]. The most commonly used search algorithms in molecular docking softwares are: Monte Carlo [21], Lamarckian Genetic Algorithm [18],[22], Differential Evolution Algorithm [15],[23],[24], Fast Shape Matching [25], and Simulated Annealing [26]. All docking softwares are implemented by search algorithms developed in order to obtain a fast computational method, capable of identifying the probable binding modes between two or more molecules [1].

Scoring Functions are able to classify the affinity or free energy of intermolecular bonding from the hypothesis that the lowest score energy represents the best orientation after the docking simulation [7]. An example of scoring function is based on a piecewise linear potential (PLP), a simplified potential whose parameters are fit to protein–ligand structures and binding data scoring functions [15], is defined by equation 1:

$$E_{score}=E_{inter}+E_{intra}$$ Eq.1

Equation 1 calculates the energies involved during the docking of ligands within a molecular target, where E_inter_ (equation 2) is the protein-ligand interaction energy, and equation 3) is internal energy of the ligand.

$$E_{inter}=\underset{i\varepsilon ligand}{\sum }\underset{j\varepsilon protein}{\sum }\left[E_{PLP}\left(r_{ij}\right)+332.0\;\frac{qiqj}{4r_{ij}^{2}}\right]$$ Eq.2

According to equation 2, the first term represents the piecewise linear potential (E_PLP_), which uses an approximation of the steric term (van der Waals) among atoms, and another stronger potential for hydrogen bonds. The second term describes a Coulomb potential with a distance-dependent dielectric constant given by: D(r) = 4r. The numerical value of 332.0 adjusts the units of the electrostatic energy to kilocalories per mole [7],[15],[24],[27].

$$E_{intra}=\underset{i\varepsilon ligand}{\sum }\underset{j\varepsilon ligand}{\sum }E_{PLP}(r_{ij})+\underset{flexiblebonds}{\sum }A[1-\;cos\left(m.\theta -\theta_{0}\right)]+\;E_{clash}$$ Eq.3

In equation 3, the first term (double summation) is among all atoms pairs in the ligand, excluding atoms pairs which are connected by two bonds. The second term is a torsional energy term, where θ is the torsional angle of the bond. The last term, E_clash_, assigns a penalty of 1000 if the distance between two heavy atoms (more than two bonds apart) is less than 2.0 Å, ignoring unfeasible ligand conformations [7],[15],[24],[27].

Currently, docking methods are accompanied by other computational techniques, such as Molecular Dynamics [28], QM/MM methods [29], and QSAR [30], providing more accuracy and reliability in the results.

### 2.2. Molecular dynamics

Molecular dynamics (MD) simulation is one of the most versatile computational methods for the study of proteins, lipid bilayers and nucleic acids [7],[31],[32]. It is a technique that provides microscopic information on the forces involved in the enzyme-ligand interaction as a function of time and also provides an understanding of the solvent's effect on protein dynamics [7],[31],[33]. Some of the most common programs used in MD studies are AMBER [34], CHARMM [35], NAMD [36],[37] and GROMACS [38].

MD methodology is based on the principles of Classical Mechanics, in which each atom is considered as a point mass, whose motion is determined by the forces acting on it by all other atoms [20]. These forces acting on each particle are calculated at each step using force fields. Force fields (equation 4) is a complete set of potential interactions among atoms, which include potential terms bound (lengths and angles of bond, dihedral angles) and unbound (interactions of van der Waals and Coulomb), a typical force field can be described by equation 4 [31],[39]:

$$\upsilon \left(r^{N}\right)=\underset{Bonds}{\sum }\frac{k_{i}}{2}{\left(r_{i}-r_{i,0}\right)}^{2}+\underset{Angles}{\sum }\frac{k_{i}}{2}{\left(\theta_{i}-\theta_{i,0}\right)}^{2}+\underset{Torsions}{\sum }V_{n}\left[1+\cos\left(n\omega -\gamma \right)\right]+\sum_{i=1}^{N}\sum_{j=1}^{N}\left(4\varepsilon_{ij}\left[{\left(\frac{\sigma_{ij}}{\sigma_{ij}}\right)}^{12}-{\left(\frac{\sigma_{ij}}{\sigma_{ij}}\right)}^{6}\right]+\frac{q_{i}q_{j}}{4\pi \varepsilon_{0}r_{ij}}\right)$$ Eq.4

Where, υ(r^N^) is the total potential energy, which is a function of positions (r) of N particles (usually atoms). The first term calculates the interactions among pairs of bound atoms, through the harmonic potential that gives the increase in energy when its length r*i* deviates from the reference value r*_i_,_0_*. The second term is a sum over all valence angles in the molecule, again calculated using a potential harmonic. The third term is the torsional potential that calculates how energy varies when the bonds spin. The fourth contribution is the unbound term, which is calculated among all pairs of atoms (i and j) which are in different molecules or in the same molecule, but separated by at least three bonds.

Some force fields include other terms; with the aim of obtaining a better agreement with vibrational spectra, for example, terms that better specify the hydrogen bonds or the coupling of oscillations between angles and bonding lengths. The choice of the force field usually depends on the properties which will be studied in the system, being a very important step in the MD simulation, because it directly influences in the reliability of the results [31].

Once the force field is defined, we can obtain the speeds whose integration allows the coordinate change of each particle. With the new speeds and coordinates of each atom, we obtain the potential and kinetic energy of the system. Therefore, it can be said that the MD simulations consist of the numerical resolution of Newton's equation of Motion, where each atom *i* of the molecular system is represented in equation 5 [31],[39].

$${\text{F}}_{i}={\text{m}}_{i}.{\text{a}}_{i}$$ Eq.5

Where F*_i_* is the force that acts on each atom of the system and a*_i_* is the acceleration of the atom *i* of mass m*_i_*. The traditional treatment only calculates the resolution of these equations for systems with at most two independent particles. Therefore, for larger systems, it is necessary the use of numerical methods and additional approaches to minimize the complexity of global force ratings. If the position in time t is r (t), the position after a short time, Δt, can be obtained following the Taylor series (equation 6) [7], as follows:

$$\text{r}\left(\text{t}+\Delta \text{t}\right)=\text{r}\left(\text{t}\right)+\frac{\text{dr}}{\text{dt}}\Delta \text{t}+\frac{{\text{d}}^{2}{}_{\text{r}}}{{\text{dt}}^{2}}\frac{\Delta {\text{t}}^{2}}{2}+\ldots$$

The solution of the equation depends on the position r (t), of speed $\frac{dr}{dt}$ and of acceleration ${\frac{dr}{dt}}^{2}$ for each particle. The time interval, Δt, of each step is an important parameter in a MD simulation. The Δt should be very small so that the acceleration can be considered constant in this range, but at very short intervals would make the calculation of the full path become prohibitive. So, the Δt used generally varies from 0.5 to 1.0 femtoseconds to provide properly the oscillations of hydrogen bonds [7],[40].

By using the MD calculations, it is possible to analyze the conformational changes suffered by the enzymes, in order to investigate and understand the behavior of a chemically active molecule in the active site of a receptor, among other processes [41].

Ochoa et al. [42], investigated reactivators for the enzyme AChE, whose main interest was to provide structural characteristics that collaborate with the proposal of new more effective antidotes for OP poisoning. To carry out this research, the authors applied some theoretical methodologies, among which the use of molecular dynamics, to analyze the dynamic behavior of the catalytic pocket of the human AChE enzyme, with the aim of providing additional clues for the design of new drugs [42].

### 2.3. QM/MM calculations

In the last decades, calculations of quantum mechanics (QM) combined with calculations of molecular mechanics (MM) became the method of choice for reactions involving biomolecular systems, solid state systems and chemical processes that occur in explicit solvents. Because these coupled techniques (QM and MM) represent a computational method that allows to model the reactions inside the enzymes associating the power and the flexibility of a quantum calculation with the simplicity of the molecular mechanics [43],[44],[45].

QM methods allow the modeling of electronic processes involved in the breaking and formation of chemical bonds, the transfer of charge, and the electrons excitation. In addition, it has the advantage of providing energy information that controls all chemical processes [44],[46]. However, QM calculations include only systems of up to a few hundred atoms, thus, the size and conformational complexity of large molecular structures is a problem for this type of calculation, since these biomolecules require methods able to analyze up to 100.000 atoms and allow simulations over scales of tens of nanoseconds [46]. One way to solve this question is to use methods of molecular mechanics (MM).

Therefore, by associating the two QM and MM techniques, it is possible to study large biomolecules and investigate a small portion of the system using quantum calculations (QM), and the rest of the system (environment) uses methods of molecular mechanics [7],[46],[47]. For example, when it comes to enzymes, the region of the active site would be analyzed by QM calculations, at an *ab initio* or semi-empirical level, or the much used density functional theory (DFT). The region of the enzyme which is not directly involved in the reaction would be treated by calculations of molecular mechanics that are based on force fields [7],[47].

One of the main characteristics among QM/MM methods is how the interactions between the boundaries of the QM and MM systems are handled, that is, the coupling of the two regions. The simplest strategy is the use of point charges in the QM region interacting with the rest of the MM region [7],[46].

Thus, in this kind of calculation, the energy of the whole QM/MM system is represented by equation 7, which indicates that all interactions between the QM/MM systems are maintained at the MM level. This is suitable for the van der Waals interactions, because QM calculations describe with little precision interactions of this type, however, it is more discussed for electrostatic interactions, since they generally provide the dominant catalytic effect [44].

$${\text{E}}_{\text{TOTAL }}{}^{\text{QM}/\text{MM}}={\text{E}}_{\text{TOTAL}}{}^{\text{MM}}+{\text{E}}_{\text{QM region}}{}^{\text{QM}}-{\text{E}}_{\text{QM region}}{}^{\text{MM}}$$ Eq.7

Where in the first term of the equation represents the MM energy of the whole system, the second term represents the QM energy of the QM region, and the last term is the MM energy of the isolated QM region. Most of the QM/MM methods consider the polarization effect in the QM region by the MM environment. These kinds of methods are associated with electrostatic interactions present in the QM and MM regions [7],[45].

The total energy of the whole system cannot be written only as the sum of the energies of the subsystems, because depending on the type of QM/MM scheme, it may include additional atoms that are part of the QM subsystem and are not part of the entire system, or they may contain atoms with special characteristics that appear in both QM and MM subsystems. Therefore, it is very important to take into account the coupling methods of the two regions at the boundary between them [7],[46].

QM/MM methods, in the enzymology fields, can be applied in order to assist in the complete understanding of enzymatic reactions, in the catalytic effect and interference of some mutations [1],[5],[48],[49].

In 2015, Giacoppo and cowokers [28] applied the methodologies of Quantum Mechanics/Molecular Mechanics (QM/MM), along with molecular docking to evaluate the reactivation kinetic constants and the interactions of the BI-6 oxime with AChE, the enzyme responsible for the hydrolysis of the Acetylcholine (ACh) neurotransmitter and the termination of the nerve impulses transmission, inhibited by different OP, in comparison to in vitro data. The results confirmed that this method was appropriate for the prediction of kinetic and thermodynamic parameters of oximes, being useful in the design and selection of new and more effective oximes.

## 3. Results

### 3.1. Organophosphorus compounds

#### 3.1.1. Chemical warfare agents and agrochemicals

During World War I, many nations developed intensive chemical warfare programs in order to improve their fighting performance. After the War, these activities were intensified even during World War II, mainly in obtaining nerve agents [50].

Nerve agents are the name of organic compounds containing phosphorus (esters of phosphonic or phosphoric acid), such as some insecticides, flame retardants, plasticizers among others. These agents in contact with the body of the victim attacks the central nervous system, then deregulating properly functioning and may leading to death [51].

Researches on the nervous agents during World War II were conducted in English laboratories and in the USA, focused mainly in the diisopropylfluorophosphate (DFP) as one of the most prominent compound. Only after the Second War the nerve agents gained military significance. Sarin gas was stored by the USA and USSR—the former Soviet Union, in large quantities, thousands of tons, along with large quantities of Soman. In addition to military laboratories, agricultural industries were very interested in OP due to their ability to combat pests [51].

In 1955, as a result of investigations by British and UK laboratories, the new class V nerve agents appeared. The VX substance was chosen as the most promising of this class and its high-scale production began in 1961 in the USA. Until today it is the most effective chemical warfare agent ever produced. The lethal dose for humans is about 0.3 mg/person after inhalation and 5 mg/person after dermal exposure. Sarin and VX have become the standard nervous agents in the USA [51].

**Figure 1.**
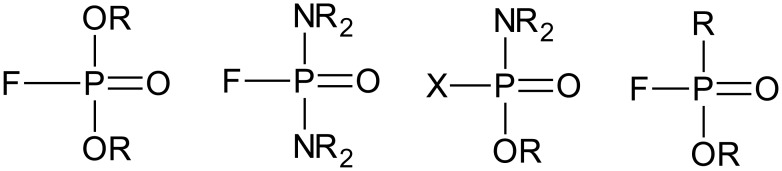
Basic structures of neurotoxic OP.

According to Jang and collaborators (2015), among synthetic chemical compounds the nervous agents are considered the most harmful ever produced. They are compounds that actively act on AChE, differing from other agents acting on blood, which cause tearing and vomiting because of their mode of action. Their chemical structure is responsible for the high toxicity in mammals compared to similar species [52]. The chemical structure of the nerve agents comprises phosphorus compounds V with one terminal oxide and three singly attached substituents (two alkoxy groups and one alkyl group) [53], as depicted in Figure 1.

The term organophosphorus refers to the group of organic compounds that contain the phosphorus element. Over the years, hundreds of these compounds have been synthesized, many of which are currently available. Several works have been performed for the development of new OP, however many results may not be revealed because of their secret objectives [54].

OP are chemical compounds that have many applications, but they are more commonly used as pesticides and chemical weapons. Currently in agriculture, pesticides such as diazinon, parathion and paraoxon are widely used to protect crops from pests and insects. However, they are highly toxic compounds, which can cause adverse effects to human health, affecting the central nervous system and then resulting in neuromuscular abnormalities that can lead to death [55]. Neuroscience research seeks to understand the impacts of nerve agents and how they can affect humans, these data can be obtained through a variety of disciplines related to chemistry, pharmacology, military warfare, biology, and medicine [53].

Most nerve agents have a common mechanism of AChE inhibition, which causes similar symptoms among the compounds of different classes. It is important to understand that there is a wide range of toxicity in these agents, making specific agent identification quite important [56].

For many reasons, a precise diagnosis and treatment for intoxication by these agents presents a challenge for clinicians. As many diseases associated with environmental exposure, pesticide poisoning remains difficult to be diagnosed, largely due to the barriers imposed by intoxication [56].

#### 3.1.2. Toxicology

**Figure 2.**
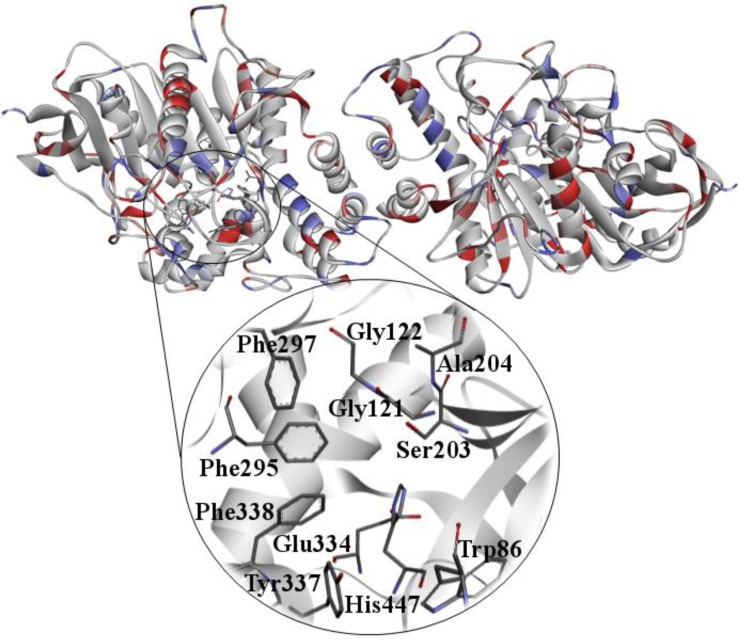
*Hss*AChE obtained from the PDB (Protein Data Bank). PDB ID: 3LII [59].

Many OP are neurotoxic and if not treated immediately after exposure, they can irreversibly inhibit AChE (Figure 2). AChE is the enzyme responsible for the hydrolysis of ACh, which terminates the transmission of nerve impulses. For this reason, it is classified as a hydrolase serine. The amino acids serine, histidine and glutamic acid constitute the catalytic triad of the enzyme [57]. This enzyme is essential for controlling the transmission of nerve impulses from nerve fibers, which influences the functioning of the organism and the central nervous system. Since a proportion of the enzyme is inactivated by phosphorylation, the signs and symptoms of cholinergic poisoning become evident [7],[58].

The loss of the enzyme function in a sufficient dosage allows the accumulation of ACh in the neurocholinergic junctions (muscarinic effects), skeletal muscle nerve junctions and autonomic ganglia (nicotinic effects). In cholinergic nerve junctions with smooth muscle and secretory cells, high concentration of ACh causes muscle contraction and secretion, respectively [60]. In the skeletal muscle junctions, the excess of ACh may be excitatory, which causes muscle spasms and may also weaken or paralyze cells by depolarizing the endplate. The involvement of the diaphragm and thoracic skeletal muscles can cause respiratory paralysis. High ACh concentration in the central nervous system causes sensory and behavioral disturbances, motor function and respiratory depression [56].

One of the main signs of OP poisoning is the overstimulation of structures innervated by cholinergic fibers (heart, glands, smooth muscles), manifesting symptoms such as miosis, salivation, bronchial, bronchoconstriction, fasciculation, convulsions and muscle weakness, culminating in death [61],[62],[63]. When nerve impulse reaches the end of the nerve, ACh is released from the presynaptic nerves into the synaptic or neuromuscular junction and binds to the ACh neurotransmitter on the postsynaptic membrane, thereby causing stimulation of nerves or muscle fibers. The acetylated enzyme allows the nucleophilic attack by water. The deacetylation reaction results in the free enzyme and the release of choline and inactive acetic acid [53],[64].

**Figure 3.**
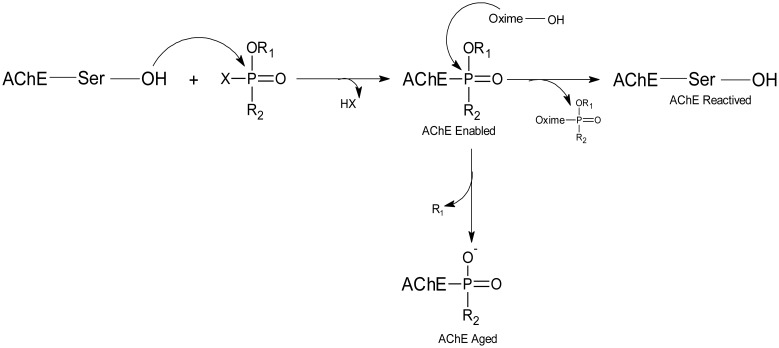
Scheme of AChE inhibition and reactivation process.

After inhibition by OP, two processes can take place involving AChE, one is aging and the other is the spontaneous reactivation of the enzyme, which can be accelerated by the addition of a strong nucleophile, such as an oxime. Without these antidotes, the reactivation reaction in most OP-AChE complexes occurs at insignificant rates, so the reaction follows the aging process [58],[65]. By dephosphorylation of the serine residue, the enzyme inhibition process can be reversed prior to aging, as shown in Figure 3.

The process of the AChE inhibition by OP is similar to the reaction with ACh, except for the step in which the leaving group of the OP is lost, so that the enzyme is phosphorylated rather than acetylated [64].

The most efficient way for the intoxication by OP takes place is via inhalation or ingestion. Penetration through skin and consequent systemic absorption can vary depending on the agents. Actually, absorption by such routes can also vary considerably. According to DuBOIS (1971) and Pasquet et al. (1976), oral administration in Parathion rats at doses of 3 to 8 mg/kg is equivalent to the 8 mg/kg dermal absorption of the same compound, which is very toxic. For the Phosalone compound, the dermal toxicity is inferior to the oral route in rats, 1500 mg/kg and 120 mg/kg, respectively [66],[67]. Highly toxic agents are more susceptible to dermal intoxication than moderately toxic agents [67].

Some OP can cause a different type of neurotoxicity, which damages peripheral and central nerve fibers associated with the Neuropathy Target Esterase (NTE) inhibition. Some compounds can accumulate in adipose tissue, which prolongs the need for use of antidotes for several days, as the stored compound is released back into the circulation. This syndrome is called Organophosphorus-Induced Delayed Neuropathy (OPIDN), manifested mainly by weakness or paralysis of the extremities, affects the legs and may persist for weeks or even years [68]. Only a few OP among the most commonly used are responsible for causing delayed neuropathy in humans. The Environmental Protection Agency (EPA) guidelines require OP and carbamate compounds to be tested on animal species susceptible to this neurotoxic property [68].

Depending on the mode of exposure to OP, symptoms of acute intoxication may occur during or after exposure. The duration of symptoms is determined by the properties of the compound: its lipid solubility, the binding stability between OP and AChE and if aging process occurs [50]. The diagnosis of acute intoxication is made from the history of exposure and the characteristic signs and symptoms. Plasma levels of pseudocolinesterase and AChE of red blood cells can be measured in blood samples, low levels of plasma pseudocolinesterase and enzymatic activity of AChE are biochemical indicators of excessive absorption of OP [50].

#### 3.1.3. Reactivation processes: oximes

**Figure 4.**
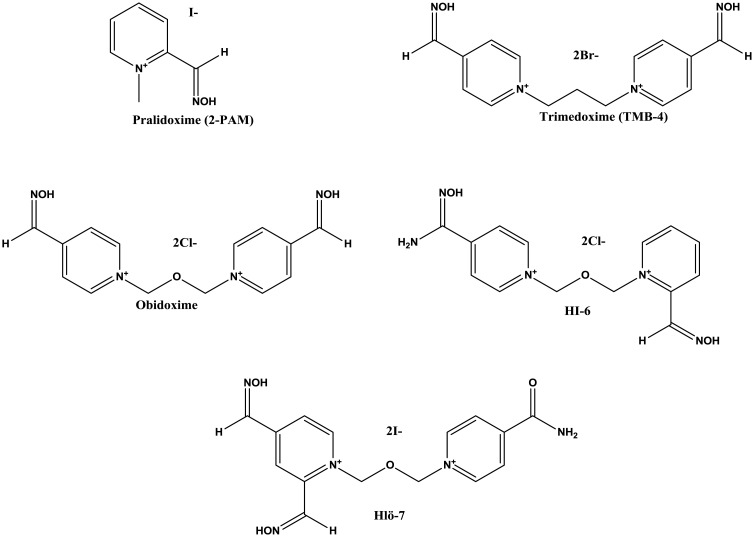
Chemical structures of pralidoxime, obidoxime, HI-6 and HLö-7.

The treatment for OP poisoning consists in the removal of the phosphoryl group from the AChE active site through the reactivation by oximes, in combination with anti-cholinergic and anticonvulsants [10]. Currently, five pyridinium Oximes (pralidoxime, trimedoxime, obidoxime, HI-6 and HLö-7) (Figure 4) are available for the clinical treatment of OP poisoning [57],[69].

However, at present, none of the substances has any spectrum of effective activity considering the differentiated neurotoxic agents, being the best efficiency associated to specific OP compounds. In this line, there is the stimulus of synthesis and analysis of more efficient derivatives for the AChE reactivation [70].

The inhibition does not reach the anionic site of AChE, further contributing to reactivation, given the binding to the cationic part of the reactivator (for example: 2-PAM. The enzyme reactivation is achieved from the oxime action and, consequently, the breakup of this OP binding with the enzyme [71].

The standardized method observed in the synthesis of oximes consists in the nucleophilic addition of hydroxylamine to aldehyde or ketone using aqueous alcoholic medium as solvent [72]. The analysis of the correlation of the antidotes structure versus activity established important aspects to benefit a good reactivation, highlighting among them the presence of quaternized ring-pyridine, the existing number of rings, the structural position presented by the oxime group (*ortho* or *para*) and the size of the spacer between the pyridine rings in the bipyridinium derivatives [73],[74],[75].

The aromatic aldehydes can lead to isomeric oximes with Z and E configurations that can be differentiated through chemical displacements of the imine hydrogens (RCH=NOH) that suffer from the shielding caused by the electron pair of nitrogen, being that the E isomer presents a chemical displacement larger than the Z isomer [72]. The search for a universal antidote against all types of OP poisoning has been a constant search in several research groups [76]. The pharmaceutical, food, agricultural and environmental areas are also interested in the AChE inhibition or reactivation [57]. Computational methods provide efficient possibilities to investigate enzymatic activity, with detailed data on the central processes of catalysis [7].

### 3.2. Enzymatic biodegradation

#### 3.2.1. Phosphotriesterase

OP compounds are transformed by a diversity of enzymes distributed along the phylogenic tree, from bacteria to mammals. Phosphotriesterase (PTE) enzyme from soil bacteria, Pseudomonas diminuta and Flavobacterium *sp*, has presented potential to degrade a wide range of OP triesters, cleaving P–O, P–F, P–CN, and P–S bonds with significant performance [77],[78],[79]. PTE is familiar due to its ability to catalyze the detoxification of chemical warfare agents, including the OP pesticides that are intensively employed in agriculture nowadays [80].

PTE (also identified in the literature as organophosphorus hydrolase—OPH) is encoded by the organophosphorus-degrading (opd) gene and takes part of the amidohydrolase superfamily [81],[82]. They share similar homodimeric structure, formed by a binuclear metal center embedded within a (α/β) 8-barrel structure. All phosphotriesterases are metal-dependent hydrolases, i.e., there is a requirement for a divalent metal, which directly binds to the substrate to favor the catalysis process [83].

**Figure 5.**
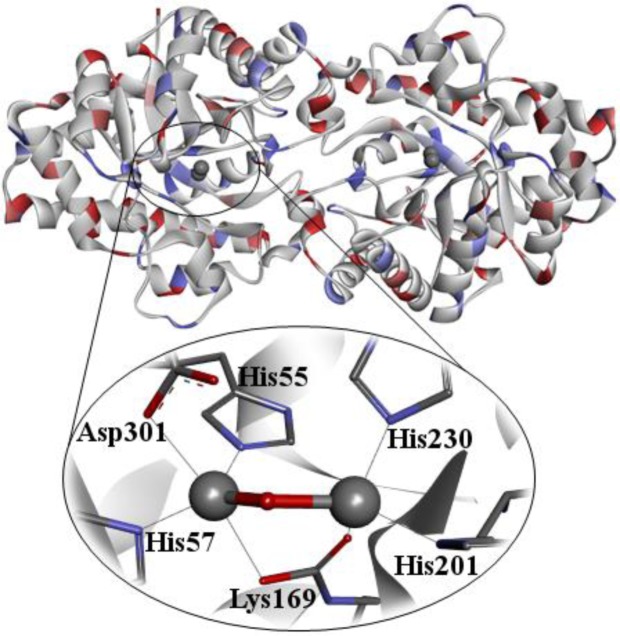
Crystallographic structure of PTE (PDB ID: 1DPM) [86].

The wild-type contains two Zn^2+^ ions that can be replaced for Cd^2+^, Co^2+^, Ni^2+^, or Mn^2+^, without loss of catalytic activity [78]. The cobalt-substituted enzyme is the most active form, though it is the most sensitive one to metal chelators [78],[84]. The two zinc cations are situated at a distance of ∼3.4 Å from each other. The more solvent-shielded Zn^2+^ ion is coordinated to His55, His57, and Asp 301 and the more solvent-exposed Zn^2+^ ion is coordinated to His201, His230, and some water molecules from the solvent, which seem to be nucleophilic species in the hydrolysis reaction performing (Figure 5) [7],[85]. While the protein folds are pretty diverse, the substrate binding sites are usually hydrophobic and can be divided into three binding pockets where the leaving group and the two other substituents interact to orientate the phosphoric center for catalysis [83]. The activation of the substrate in the phosphoric center is achieved by a direct interaction between the phosphoryl oxygen and a divalent metal in the active site [83].

The OP inactivation is of extreme significance, given that the accumulation of these agents leads to AChE inhibition [87], which is a key enzyme responsible for the nerve functions in human beings. In this line, PTE hydrolyses these toxic agents (triesters), giving rise to those less toxic (diesters) [81]. Wild-type PTE is known for its property of being stereoselective in the degradation of chiral substrates [88], with the stereoselectivity degree depending on the substituents attached to the phosphoric core [89],[90]. For instance, wild-type PTE degrades the R_P_-enantiomer of phenyl 4-acetylphenyl methylphosphonate about two orders of magnitude faster than for the S_P_-enantiomer [90]. The chiral enantiomers of OP differ largely in relation to their acute toxicity [1]. Thus, the relative stereospecificities of PTE is quite necessary not only to fully comprehend the chiral nature of OP nerve agents reaction with this enzyme, but also to design more efficient enzymes for detoxification of these compounds [91]. The intrinsic stereoselectivity of the wild-type PTE does not match that of AChE, due the fact of PTE preferentially degrades the least toxic enantiomer of chiral OP (R_P_-enantiomer), but on the other hand, the PTE stereoselectivity is susceptible to manipulation through the mutation of residues that contact the substrate prior to catalytic turnover [92].

**Figure 6.**
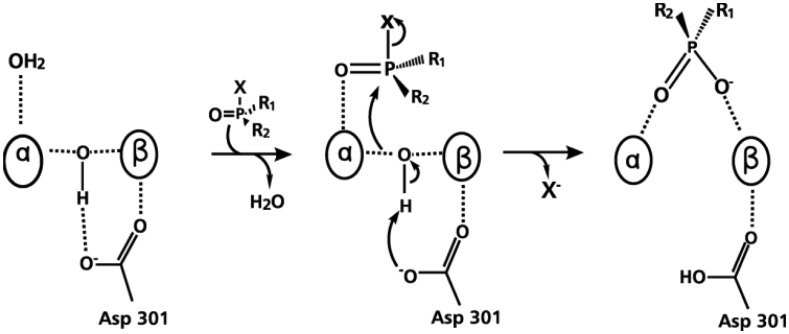
Proposed hydrolysis for OP (α/β = Zn^2+^) by PTE [94].

The proposed reaction mechanism for the PTE catalyzed reaction begins with a nucleophilic attack by an activated water molecule on the phosphoric center, resulting in an inversion of configuration. That is, the reaction takes place via bimolecular nucleophilic substitution (S_N_2) mechanism, where the Asp301 serves as a weak base that removes a hydrogen atom of the water molecule, activating it. Afterwards, the resulting hydroxyl ion attacks the central P (Figure 6) [91],[93].

A deeper understanding of the enzymatic hydrolysis mechanism of PTE is necessary to explore new insights in the bioremediation process of OP. This catalytic reaction mechanism has been widely investigated [83]. From the reaction mechanism suggested in Figure 6, both Zn^2+^ cations in the active site are immediately involved in the hydrolysis. The attack of the hydroxyl ion on the P takes place with the leaving group being released from the substrate. As described previously, this reaction is assisted by Asp301 [83],[94],[95].

Several crystallographic structures have been deposited for the wild-type PTE (PDB ID: 1DPM; resolution = 2.10 Å) [86] and its mutants (PDB ID: 2OQL, 3UR5, etc.). These structures have stimulated more studies on the substrate affinity and reaction mechanism, especially computational investigations [1],[83]. These studies have revealed important contributions for the understanding of OP hydrolysis mechanism by PTE, and have suggested new possibilities to design more effective PTE mutants.

Some interesting works by using theoretical methods in the study of biological systems (PTE enzyme, for example) are found in literature. For instance, Chen et al. [85] employed quantum chemical modeling to study enzymatic reactions of PTE in the degradation of OP triesters. The researchers made an assessment about the adequation of some technical approaches commonly employed in the modeling of enzymatic reactions with high level methodologies, including the choice of basis set for geometry optimizations and energy assessment, the choice of dielectric constant to model the enzyme environment, and the effects of blocking the centers of truncation [85]. In a study from Perezgasga et al. [79], they have replaced Zn^2+^ ions with Co^2+^ ions, and observed a significant increase in the catalytic activity of PTE with different OP pesticides. In this case, QM/MM calculations were carried out [79].

#### 3.2.2. Human serum paraoxonase 1

As seen so far, enzymatic treatment has become an innovative, robust and effective alternative for removing toxic agents, such as the neurotoxic OP [96]. Substantially, these species known for “green catalysts” are able to perform distinct reactions very frequently at an elevated rate, which is not possible to be reached by the traditional chemical catalysis [96],[97]. Previously, similar to PTE, some degrading enzymes were characterized and the human serum paraoxonase 1 (*Hss*PON1) has shown good potential for enzymatic catalysis in the OP degradation [96].

**Figure 7.**
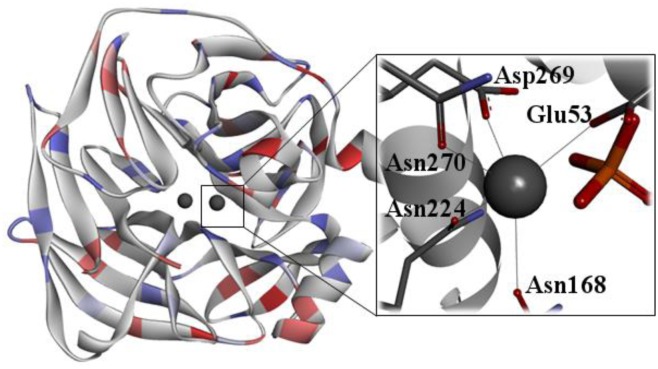
Crystallographic structure of PON1 (PDB ID: 1V04) [113].

*Hss*PON1 is able to degrade a wide range of organic esters and neurotoxic OP, which is of significant interest in the medicinal area [97]. For example, this enzyme is capable of degrading pesticides such as paraoxon, and also the lethal nerve agents such as VX, Sarin, Tabun, Soman, among others [98]–[103]. However, the wild-type *Hss*PON1 (Figure 7) presents a lower catalytic efficiency than that necessary for significant and efficient protection against intoxication by OP [104]. In this line, many mutants designed from the wild-type *Hss*PON1 have been proposed with the purpose of improving its catalytic efficiency [100],[102],[103],[104]. *Hss*PON1 is a Ca^2+^-dependent serum enzyme whose exact physiological functions are still unknown [105],[106]. However, this enzyme presents some significant properties, such as anti-inflammatory, anti-oxidative, etc [106]–[111]. The *Hss*PON1 is synthesized in the liver and it is characterized by being bonded to high-density lipoproteins (HDLs) in the bloodstream [112].

Actually, the crystal structure of *Hss*PON1 has not been elucidated, but high-resolution serum paraoxonase structures have been determined by X-ray crystallography for other organisms [113]. An initial step to a better comprehension of the hydrolysis of OP by *Hss*PON1 is the determination of the three-dimensional structure of this enzyme. Currently, there is no experimental structure of *Hss*PON1 available. However, a high-resolution structure of PON1 from *Rattus rattus* (*Rr*PON1) has been described through X-ray crystallography (PDB ID: 1V04; resolution = 2.20 Å) [113]. The 83.7% sequence identity between *Rr*PON1 and *Hss*PON1 makes it an excellent template for the design of a 3D model for *Hss*PON1 [114]. The structure of this enzyme presents two Ca^2+^ ions and a phosphate group, localized at the active site [113]. One Ca^2+^ ion with structural function is responsible for maintaining the enzyme structure, and the other is directly involved in the catalytic activity [115]. Due to the lack of a complete crystal structure, there is a doubt about the reaction mechanism which *Hss*PON1 follows. Thus, the comprehension of the *Hss*PON1 in relation to the substrate recognition mechanism is necessary to detail the enzyme-catalyzed reactions in the OP biodegradation process, which can be employed as bases for the designing of mutant enzymes with a better degrading catalytic activity [100],[102],[103],[104].

One approach to better understand the exact mechanism in which *Hss*PON1 degrades OP is to determine how the enzyme stabilizes the important chemical intermediates in the reaction pathway. In line with that, we put in evidence three possible potent reaction mechanisms for *Hss*PON1 in the degradation of OP nerve agents [97],[99],[102],[103],[116]–[119].

**Figure 8.**
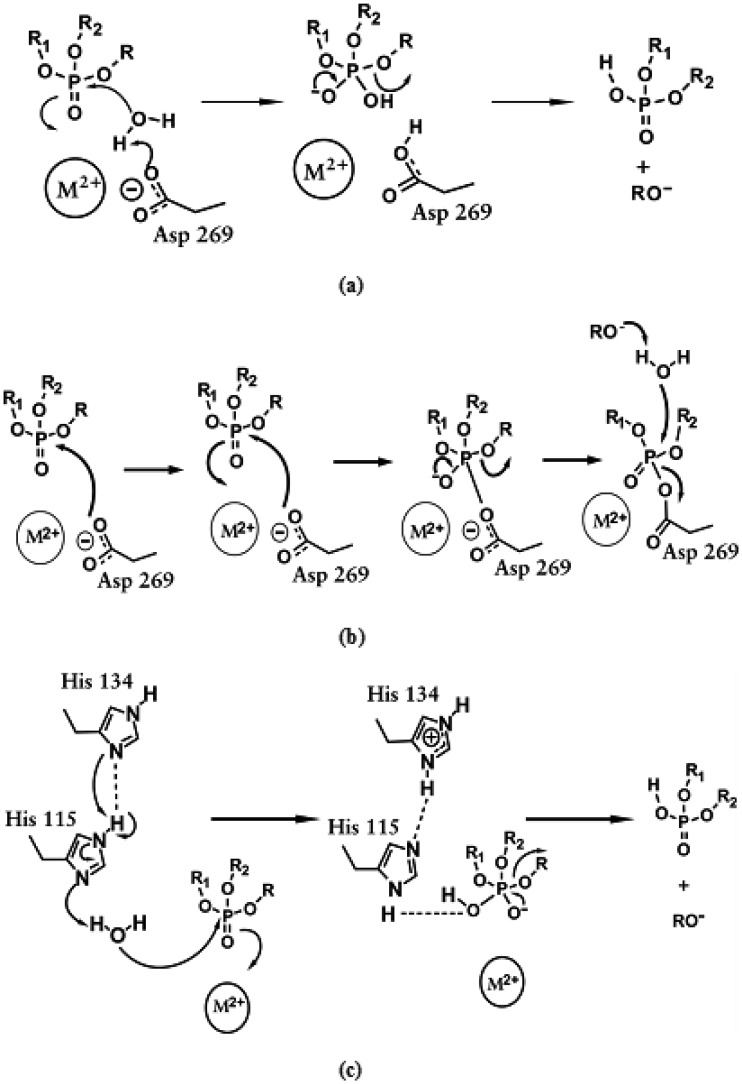
Proposed reaction mechanisms for the hydrolysis of OP by *Hss*PON1 and related mutants (M = Ca). Mechanisms (a) and (b) were described by Worek et al. [103],[119] and mechanism (c) was described by Tawfik et al. [119].

According to previous studies, the first reaction detailed here consists in the activation of a water molecule, due to the direct coordination with the catalytic Ca^2+^ cation and elimination of a hydrogen atom from the water molecule by Asp269 amino acid residue, resulting in the formation of a hydroxyl ion. This activated water directly attacks the OP, more precisely on the central P, with the expulsion of the leaving group (Figure 8a) [94],[103],[120]. This first mechanism reported here is similar to that followed by PTE in the OP degradation process [94],[120].

For the other potential reactions, no water molecule is necessary, because there is an attack on the phosphoric core immediately by the Asp269 amino acid residue, also promoting the expulsion of the leaving group and cleavage of the compound (Figure 8b) [103]. Finally, the last possible mechanism is related to the participation of His134 and His115 amino acid residues in the water activation step, for the subsequent attack to the OP (Figure 8c) [97]. It is proposed that the OP degradation mechanism by the wild-type *Hss*PON1 involves a higher reaction rate in relation to a preferential enantiomer. Probably, the same tendency could be applied to its mutants.

Some computational studies have been developed with *Hss*PON1, for example, we can cite the work from Muthukrishnan et al. [121], whose research consisted in an evaluation of the substrate tolerance in a promiscuous enzyme. Their findings imply that while *Hss*PON1 is a promiscuous enzyme, there are considerable constraints in the active site pocket, which could be related to both the leaving group and the retained portion of paraoxon analogs [121]. Another interesting work is a contribution from Fairchild et al. [114], which is related to a computational assessment and characterization, investigating how VX binds *Hss*PON1. Their results show that the lone oxygen atom in VX structure presents a high preference for creating a direct electrostatic interaction with Ca^2+^ ion in the enzyme active site. The results from the binding simulations also show that steered molecular dynamics can strongly be employed to get more precise binding predictions, even when starting with a closed conformation of a protein's binding or active site [114]. Sanan et al. [119], described the design of protein templates for the human isoform obtained from a crystal structure of a chimeric version of the protein (PDB ID: 1V04) and a homology model obtained from the related enzyme diisopropyl fluorophosphatase (PDB ID: 1XHR), providing significant contributions for the computational enzymatic catalysis [119].

#### 3.2.3. Diisopropyl fluorophosphatase

The study of enzymes capable of degrading neurotoxic OP has become the focus of several researches in several countries, in relation to the potentiality and high toxicity of these compounds. The use of Diisopropyl fluorophosphatase (DFPase) is promising for enzymatic detoxification due to its thermal stability and high tolerance to organic solvents [122]–[125]. One of the early works on this enzyme was published in 1966, where an unexpected hydrolysis activity against the Diisopropyl fluorophosphate (DFP) was found in the axoplasm of squid axon [126]. Since then, several studies on wild-type DFPase and hydrolysis activity have been performed [127]–[134]. However, its native function remains uncertain [122].

DFPase, from *Loligo vulgaris*, is a Ca^2+^-dependent phosphotriesterase that has been considered potent biocatalysts for the hydrolysis of a range of highly toxic OP compounds, including several nerve agents. In contrast with DFP substrate, the neurotoxic agents possess an asymmetric P atom, which leads to pairs of enantiomers that exhibit different toxicities [135]. In this context, it was reported that DFPase is stereoselective for the least toxic stereoisomer of the G-type nerve agents, usually R_p_-enantiomer [135]. Furthermore, the enzymatic detoxification reaction of neurotoxic leads to the formation of phosphate or phosphonate, fluoride ions, or cyanide ions in the Tabun degradation [122]–[125].

**Figure 9.**
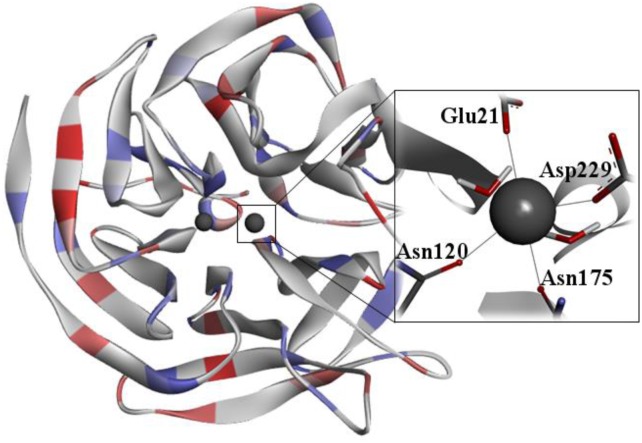
3D structure of squid DFPase (PDB ID: 2GVV) [134].

DFPase has 314 amino acid residues and adopts a fold structure of six blades in β-helix. Each blade consists of four twisted antiparallel β sheets, which form a central water tunnel with two Ca^2+^ ions. On one hand, the Ca^2 +^ at the bottom of the active site is required for catalytic activity. On the other hand, the Ca^2+^ situated in the center of the water tunnel is related to the enzyme structural integrity. Previous works demonstrate that the calcium ions can be replaced with other divalent metal ions (Mg^2+^, Ba^2+^, Sr^2+^, Mn^2+^, Ni^2+^, Co^2+^) without significant loss in the catalytic activity [122],[123],[125],[136]. In the enzyme active site, the catalytic Ca^2+^ is coordinated to seven ligands, three water molecules and the side chain of Glu21, Asn120, Asn175, Asp229, as shown in Figure 9 [131],[133].

Previous studies [130],[133], from X-ray and mutagenesis experiments, suggest an essential role of His287 in the DFPase catalytic activity. In the proposed mechanism, the substrate coordinates to the catalytic Ca^2+^ ion through the phosphoryl oxygen, by the displacement of a water molecule, such that the leaving group points away from His287. Subsequently, a water molecule is activated by His287 through proton abstraction, and then acts as a nucleophile on P. In the last step, the leaving group is released and the hydrolyzed substrate is removed from the Ca^2+^ ion [123],[134]. However, investigations with some His287 mutants suggest that this mechanism is probably incorrect [124]. To solve this inconsistency, Blum et al. [134] conducted theoretical/experimental studies to investigate the hydrolysis mechanism by DFPase. They describe the design and synthesis of DFPase substrates. The DFPase crystal structure with the strongest binding substrate dicyclopentylphosphoroamidate (DcPPA) was reported (PDB ID: 2GVV) [134], along with the characterization of its binding mode, via a combination of NMR, kinetic and computational techniques [134].

Structural analysis and docking results of DcPPA showed that the leaving group points to His287. This orientation does not allow the attack by hydroxide, suggesting that the hydrolysis mechanism involving the activation of water, His287-dependent, is structurally infeasible. The results observed by Blum et al. [134] also suggest that the only residue in the correct orientation to perform a nucleophilic attack is the Asp229. Based on this feature, they proposed the mechanism wherein the hydrolysis proceeds by nucleophilic attack of Asp229 on the P center. Then, the removal of the leaving group produces phosphoenzyme as intermediate.

**Figure 10.**
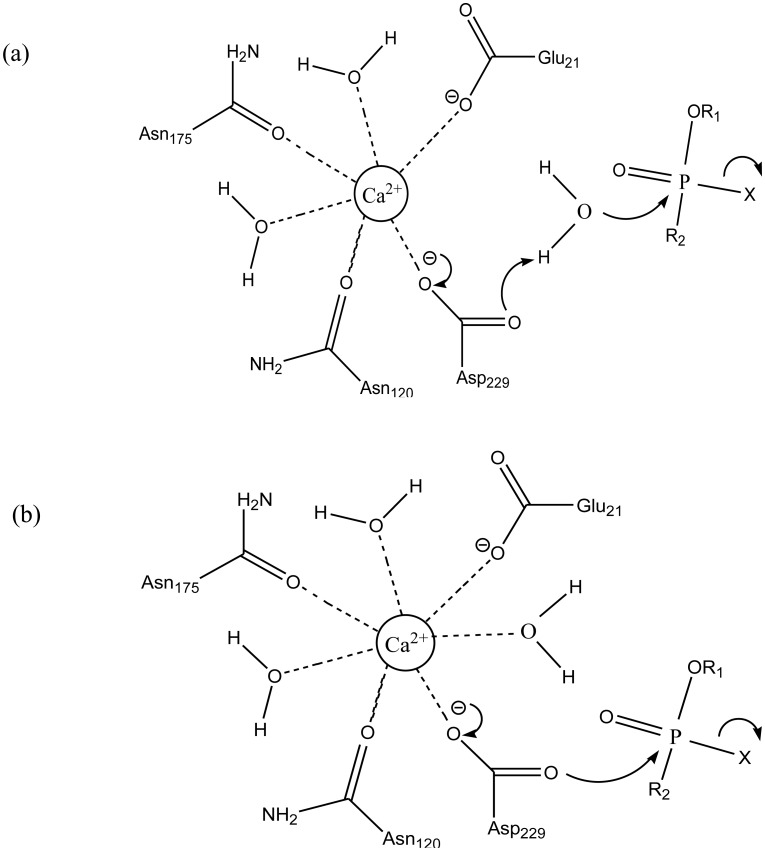
Proposed mechanisms for OP degradation by DFPase. (A) Through an activated water molecule by Asp229; (B) Through direct nucleophilic attack by Asp229 [122],[134].

Wymore et al. [122] also investigated the hydrolysis mechanism by DFPase. They employed QM/MM simulations with DFT to study the hydrolysis mechanism of DFP and S_p_-Sarin. The authors found that the DFP hydrolysis mechanism is similar to that proposed by Blum et al. [134], which takes place by nucleophilic attack from Asp229 on the P center, giving rise to a pentavalent intermediate, prior to the leaving group being released from the substrate. However, this same mechanism is energetically unfavorable for the S_p_-Sarin hydrolysis, wherein the intermediate is not observed. Instead, their results support the idea that the mechanism does not take place by direct nucleophilic attack of Asp229, but by a water molecule activated by Asp229. This may be due to the stabilization of the negative charge of the nucleophile by DFP. It was an unexpected result given the similarity in the catalytic efficiency for both substrates [122]. Figure 10 illustrates the two proposed mechanisms for the degradation of OP by DFPase.

It has been reported by Melzer et al. [135], that DFPase is stereoselective for the least toxic stereoisomer. In this context, Melzer et al. [135] developed an experimental/theoretical study which aimed to reverse the DFPase stereoselectivity to the most toxic stereoisomers of Sarin, Soman and Cyclosarin, while at the same time still hydrolyzing the least toxic enantiomer, thus ensuring a complete detoxification. However, a suitable hydrolysis orientation is found only for the least toxic enantiomer (R_p_), which allows an appropriate nucleophilic attack of Asp229 to the P center.

## 4. Conclusions

Chemical weapons represent a major concern to the society due to their devastating effect. The misuse of OP can cause serious damages to public health and the environment. Therefore, the search for efficient detoxification pathways for neurotoxic OP compounds as well as pesticides is very important. To date, the most used chemical warfare agents are OP compounds. This concern has encouraged the search for efficient tools for the detection and detoxification of neurotoxic OPs. It is well-known that conventional methods for OP detoxification are expensive, require harsh conditions, and strict containment. In this context, bioremediation processes are currently one of the most promising technologies for the OP degradation, along with the use of specific antidotes, such as oximes. As described in this review, the enzymes *Hss*PON1, DFPase and PTE have been shown to be very efficient for this purpose. Thus, in order to better investigate the action mechanisms of these enzymes, computational techniques such as Molecular docking, MD simulations and the hybrid QM/MM approach have been presented, being powerful tools able to provide important details on the microscopic behavior of the interaction of these compounds in the target enzymes. Thus, we believe that theoretical investigations are crucial for the development of more efficient techniques for the detoxification of the poisoning caused by OP.
